# Neurodevelopmental Trajectories of Preterm Infants of Italian Native-Born and Migrant Mothers and Role of Neonatal Feeding

**DOI:** 10.3390/ijerph17124588

**Published:** 2020-06-25

**Authors:** Dino Gibertoni, Alessandra Sansavini, Silvia Savini, Chiara Locatelli, Gina Ancora, Enrica Perrone, Magda Ialonardi, Paola Rucci, Maria Pia Fantini, Giacomo Faldella, Luigi Corvaglia

**Affiliations:** 1 Department of Biomedical and Neuromotor Sciences, University of Bologna, 40126 Bologna, Italy; dino.gibertoni2@unibo.it (D.G.); enrica.perrone2@unibo.it (E.P.); magda.ialonardi@gmail.com (M.I.); paola.rucci2@unibo.it (P.R.); mariapia.fantini@unibo.it (M.P.F.); 2 Department of Psychology, University of Bologna, 40126 Bologna, Italy; silvia.savini3@unibo.it; 3 Neonatology and Neonatal Intensive Care Unit, S. Orsola-Malpighi Hospital, 40138 Bologna, Italy; chiaralocatelli77@gmail.com (C.L.); gina.ancora@auslromagna.it (G.A.); giacomo.faldella@unibo.it (G.F.); luigi.corvaglia@unibo.it (L.C.); 4 Neonatal Intensive Care Unit, Infermi Hospital Rimini, 47923 Rimini, Italy; 5 Department of Medical and Surgical Sciences, University of Bologna, 40126 Bologna, Italy

**Keywords:** preterm infants, low-birthweight, migrant mothers, neonatal feeding, neurodevelopment

## Abstract

There is evidence that preterm infants of migrant mothers are at a higher risk of adverse perinatal outcomes than those of native-born mothers, and that human milk feeding is beneficial to infants’ neurodevelopment. Using the United Nations Human Development Index (HDI) to classify mother’s country of origin, we investigated whether type of neonatal feeding (human milk vs. mixed milk vs. exclusive formula milk) affected preterm newborn neurodevelopment varying across different HDI categories (Italian native-born vs. high HDI migrant vs. low HDI migrant) up to 2 years of age. Neurodevelopment of 530 infants born in Italy at ≤32 weeks of gestational age and/or weighing <1500 g was measured at 3-, 6-, 9-, 12-, 18-, and 24-months corrected age (CA) using the revised Griffiths Mental Development Scale 0–2 years. The trajectories of the general developmental quotient and its five subscales were estimated using mixed models. At 24-months CA only preterm infants of low HDI migrant mothers and fed exclusive formula milk showed moderate neurodevelopment impairment, with lower developmental trajectories of eye-hand coordination, performance, and personal-social abilities. Migrant mothers from low HDI countries and their preterm infants should be targeted by specific programs supporting maternal environment, infant development, and human or mixed milk neonatal feeding. Future research should focus on a deeper understanding of the mechanisms through which type of feeding and mother migrant conditions interact in influencing preterm infants’ neurodevelopment.

## 1. Introduction

Preterm birth is characterized by multiple interacting biological and environmental factors that may lead to atypical developmental trajectories [1]. Neonatal immaturity, characterized by extremely or very low gestational age and birthweight, and clinical complications, such as bronchopulmonary dysplasia (BPD), severe retinopathy of prematurity, necrotizing enterocolitis (NEC), intraventricular hemorrhage, and sepsis, may contribute to motor and cognitive developmental abnormalities [2]. Developmental trajectories may also be affected by type of feeding [3]. Human milk (HM) is associated with brain development, particularly white matter [4]. In addition, HM decreases the incidence of sepsis and NEC that are associated with adverse neurodevelopment outcomes in preterm infants [5]. 

Socioeconomic factors also play an important role in neurodevelopment. It was estimated that approximately 13% of the intelligence quotient discrepancy between low- and normal-birthweight infants can be attributed to social determinants of health [6]. Infants’ feeding is related to familial socioeconomic status; specifically, conditions favoring breast feeding are less common among women with social disadvantage [7,8].

Among socioeconomic factors, migration is nowadays very relevant, as a result of the large migratory flows towards many Western countries in the last two decades, mostly originating from less-developed countries. Many migrant mothers from these areas are exposed to lower access to social support, low socioeconomic status, and lack of proficiency in the hosting country language. Minority ethnicity and refugee- or asylum-seeking status confer migrant mothers an increased risk of requiring economic and psychological support, experiencing distress and isolation [9], depressive symptoms, and adverse outcomes for both the mother and the child [10,11]. Also, malnutrition, which includes both undernutrition and overnutrition, is more frequent in low-resource settings, such as those often experienced by migrant and low socioeconomic status pregnant women [12]. As highlighted by Vohr and colleagues [12], malnutrition during gestation can impact maternal and fetal neurodevelopment and micronutrient concentration in breast milk, with long-term consequences for physical and mental health of both the mother and her child. Thus, the characteristics of the environment of the pregnant woman, including malnutrition, infections, exposure to tobacco, alcohol, drugs, etc., impact fetal and neonatal neurodevelopment, and this impact may even be greater for infants born in low-income countries where neonatal morbidity is higher and healthcare facilities are less available [13]. As for preterm infants’ neurodevelopment, a systematic review of studies including children born at a gestational age ≤32 weeks or with a birthweight ≤1250 g showed that non-white ethnicity and low parental education predict poorer cognitive development at 18- to 30-months of age [14]. 

Neurodevelopment evolves rapidly in the infants’ first years of life, and psychomotor impairment during infancy predicts impairment at a later age [15]. Thus, it is important to investigate neurodevelopment in the first two years of life, especially by means of longitudinal studies that allow a deeper understanding about the timing at which impairments appear and the interactions of risk factors. However, to date neurodevelopmental trajectories of preterm infants during early childhood have been rarely studied [16], because longitudinal studies focused mainly on childhood [17,18] and adolescence [19,20,21].

In this study we estimated developmental trajectories of preterm infants in subgroups defined by type of feeding and mothers’ country of origin from 3-months to 2-years of age. We hypothesized that human milk feeding would positively affect preterm developmental trajectories across different HDI categories even if a disadvantage for infants of less-developed countries migrant mothers was expected. This study might have relevant implications for clinical practice, because in Italy, and particularly in Emilia-Romagna, the region of north–east Italy where our study took place, deliveries from migrant mothers has increased from 17.1% of total deliveries in 2003 to 31.0% in 2015 and mothers not born in Western countries and social disadvantaged mothers are less likely to breastfeed their babies during Neonatal Intensive Care Unit (NICU) stay [22].

## 2. Materials and Methods

### 2.1. Participants and Procedures

**Figure 1:**
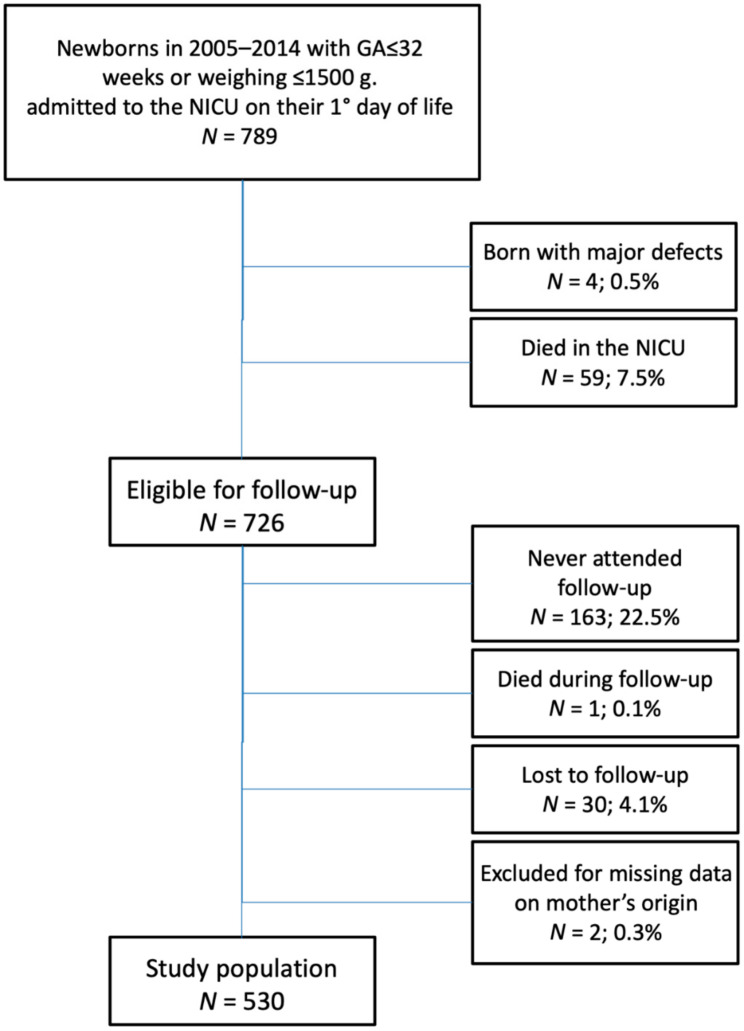
Flow chart of the study population. GA, gestational age; NICU, neonatal intensive care unit.

The population of this prospective cohort study consisted of 726 newborns with a gestational age ≤ 32 weeks and/or birthweight < 1500 g, admitted on their first day of life to the Neonatal Intensive Care Unit (NICU) of the S.Orsola-Malpighi University Hospital in Bologna, north–east Italy, from 1 January, 2005 to 31 December, 2014 and eligible for a follow-up program devised to evaluate their physical and psychomotor development up to 24-months corrected age (CA). Anthropometric and psychomotor development data were retrieved from the preterm newborns database of the NICU and were linked with the perinatal data extracted from the Vermont Oxford Network database. We excluded those who never attended follow-up visits (*n* = 163, 22.5%), died during follow-up (*n* = 1, 0.1%), were lost to follow-up (*n* = 30, 5.3%), and those for whom information on mother’s country of birth was missing (*n* = 2, 0.3%). Thus, the final study sample included 530 newborns (Figure 1). The study procedures were approved by the Bologna Health Authority’s Independent Ethics Committee (76/2013/U/Sper/AOUBo). All parents of the preterm infants gave written informed consent for participation in the study, including data analysis and publication. The datasets analyzed in the current study are not publicly available because they are included in a clinical registry with restricted use.

### 2.2. Outcome Measures

Infants were evaluated at 3-, 6-, 9-, 12-, 18-, and 24-months CA by an experienced psychologist with a master’s degree in developmental neuropsychology, blinded to the feeding status of infants. The assessment included the revised Griffiths Mental Development Scales (GMDS-R) 0–2 years [23]. This scale evaluates five developmental domains: locomotor (LOC), personal–social (PS), hearing–language (HL), eye and hand coordination (EH), performance (PERF), yielding standardized subscale quotients and a general developmental quotient (GQ). GQ was calculated using the tables of standardized scores for the English infants’ population (mean 100.5, standard deviation—SD 11.8), because standardized data for the Italian population were unavailable. Following the criteria used in studies investigating developmental outcomes [16,17,23], normal development was defined as a score GQ ≥ 88.7, and cut-offs for mild, moderate, or severe impairment were 88.6, 76.8, and 65, respectively. 

### 2.3. Socio-Demographic and Clinical Variables

Mothers’ country of origin was classified using the United Nations Human Development Index (HDI) [11,24]. HDI ranks countries according to their average achievement in three key dimensions of human development: long and healthy life, knowledge, and standard of living (measured by life expectancy at birth, years of schooling, and gross national income per capita). Using the HDI quartiles provided by United Nations, countries were grouped in two categories: high HDI (first and second quartile) and low HDI (third and fourth quartile). Italy that ranks in the first quartile, was used as reference category. The list of countries by HDI quartiles is provided in the Supplementary File Table S1.

Feeding was recorded at discharge from the NICU and coded as human milk (own mother’s raw milk, either bottle-feeding or breastfeeding), mixed (human milk > 50% of daily intake), or exclusive formula milk. Infants fed formula milk at discharge were either those fed formula milk during the entire hospitalization or those who switched to formula milk during hospitalization. Collected birth and neonatal data included: gestational age (GA) in weeks (corrected in order to take into account their level of neuropsychological maturation); small for gestational age (SGA) at birth and discharge (identified by a standardized weight at birth < −1.28, corresponding to the 10th percentile of the distribution of Italian Neonatal Anthropometric Charts [25]); and complications occurring during hospitalization, including mechanical ventilation (MV), bronchopulmonary dysplasia, early and late onset sepsis (both culture proven or clinical sepsis), necrotizing enterocolitis requiring surgery, severe intraventricular hemorrhage (IVH; grade 3 and 4 as classified by Papile et al. [26], including post-hemorrhagic hydrocephalus requiring surgery), or periventricular leukomalacia (PVL) defined as the presence of periventricular cysts at any cranial ultrasound performed during hospital stay. Mothers’ characteristics included parity and a composite index of social risk ranging from 0 to 3, adapted from Mangin et al. [19]. This index was obtained as the count of three risk factors: age < 23 or >40 years, low education (≤8 years), unemployed, or housewife. Education level was defined according to the following categorization: low (≤8 years), middle (9–13 years), and high (>13 years). Unemployed mothers were temporarily jobless women who were actively looking for a paid job, whereas housewives are women who are dedicated to family and house care not looking for a paid job. Data on maternal nutrition were not collected, and maternal body mass index (BMI) was available only for 167/530 (31.5%) cases. Smoking status comparing non-smokers to mothers who were smokers during pregnancy or in the five years preceding pregnancy was available for 423/530 (79.8%) mothers. Data on maternal alcohol use was not available. Lastly, a dichotomous variable indicating birth in the first (2005–2009) or in the second (2010–2014) five years of the study was used to capture a possible cohort effect reflecting changes in care occurred in the decade.

### 2.4. Statistical Analyses

Infant characteristics and GQ scores were compared among HDI and feeding categories using *χ*^2^ test, Fisher’s exact test, ANOVA, or Kruskal–Wallis test as appropriate. Post-hoc pairwise comparisons were performed at a Bonferroni corrected significance level of *p* = 0.017. GQ trajectories were estimated using mixed-effects regression with random intercepts and slopes, to allow individual variations in the GQ score at 3-months CA (the intercept) and in the trajectories over time (the slope). A three-way interaction of time × HDI × diet was included in the model to compare the nine trajectories of the groups defined by HDI and type of feeding. Baseline newborns clinical variables, mothers’ parity, smoking status, and the composite index of social risk were used as fixed effects and removed with a backward elimination procedure if they did not contribute significantly to the prediction of GQ trajectories. The mixed-effect model was obtained with multiple imputation of missing data using chained equations and was replicated for each of the five GMDS-R subscale scores. Stata v.15.1 (StataCorp LLC, College Station, TX, USA) was used for all analyses, specifically the mimrgns [27] and coefplot [28] user-written modules were used to plot of trajectories with 95% confidence intervals. The significance level was set at *p* = 0.05. 

## 3. Results

### 3.1. Characteristics of the Study Sample

The study population included 384 (72.5%) preterm newborns of Italian mothers, 87 (16.4%) of high HDI and 59 (11.1%) of low HDI countries migrant mothers. Diet at discharge was mixed for 238 (44.9%) newborns, exclusively formula for 150 (28.3%), and human milk only for 142 (26.8%) newborns. The comparison of mother and newborn characteristics among HDI categories (Table 1) showed that migrant mothers from a low HDI origin were significantly younger, more likely to be housewives, had a significantly lower educational levels and were less likely to be primiparous. The mother’s social risk score increased significantly from the Italian group to the high and low HDI migrant group. Maternal BMI did not differ among HDI and diet subgroups, in the subset with available data (data not shown).

Comparisons according to the type of feeding showed that formula-fed newborns had lower birthweight, were more likely to be very or extremely preterm, used more mechanical ventilation, had less educated mothers who were more frequently smokers and were less frequently twins (Table 2). The maternal risk score was significantly higher in formula-fed newborns than in newborns fed human milk.

### 3.2. Empirical Neurodevelopmental Trajectories According to HDI and Diet

**Figure 2:**
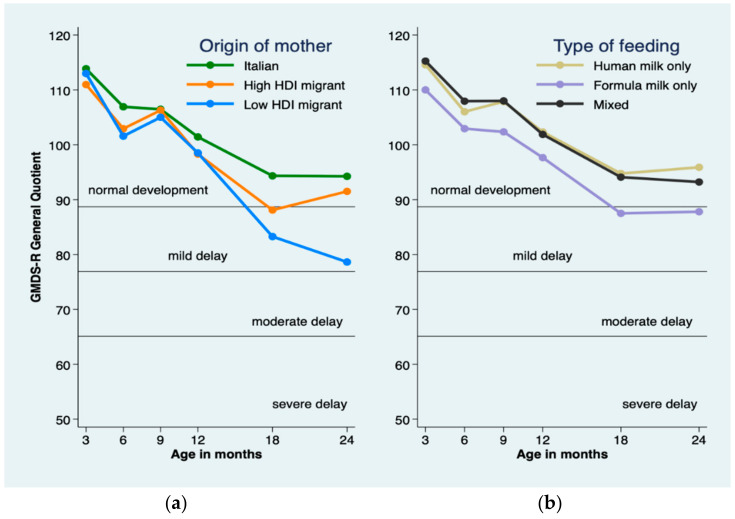
Observed trajectories of revised Griffiths Mental Development Scales (GMDS-R) general quotient by origin of mother (**a**) and by type of feeding (**b**). HDI, Human Development Index

Figure 2 shows the empirical GQ trajectories by HDI and diet. All trajectories declined over time but diverged at 18- and 24-months CA among HDI groups. Specifically, GQ scores were significantly higher in the Italian group than in the low HDI group at 18- and 24-months and in the high HDI group at 18-months (Table 3). 

As to diet, exclusively formula-fed infants had significantly lower neurodevelopment trajectories, compared with the other groups (Table 3 and Figure 2—right panel). The trajectories of neurodevelopment were generally lower in children with greater persistence of weight restriction during follow-up (Figure S1).

### 3.3. Estimated Neurodevelopmental Trajectories According to HDI and Diet

The GQ trajectories were estimated using a mixed-effects model adjusted for newborns’ and mothers’ covariates. The covariates retained in the final model, after removing non-significant ones, were mother’s smoking status, and infant IVH/PVL, MV, BPD, sepsis, and SGA at discharge. They all negatively affected GQ with coefficients ranging from −2.10 for SGA at discharge to −12.49 for IVH/PVL (see Supplementary File, Table S2 for the detailed Stata output). Notably, mother’s social risk score was not retained in the final model because, after including the HDI category, it did not account for variations in GQ trajectories.

**Figure 3:**
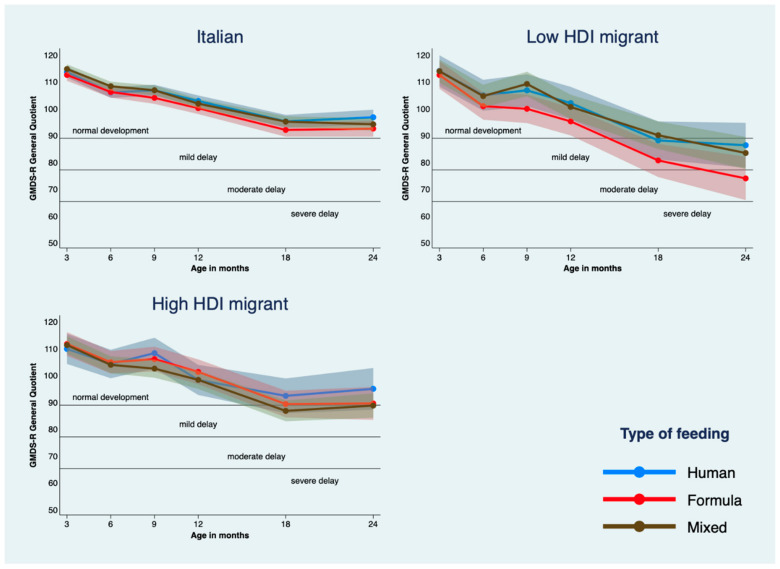
GMDS-R general quotient trajectories and 95% confidence intervals estimated by using mixed-effects modeling, by mother’s origin and type of milk feeding and adjusted for mother’s smoking status, and infant intraventricular hemorrhage or periventricular leukomalacia (IVH/PVL), sepsis, bronchopulmonary dysplasia (BPD), mechanical ventilation, and small for gestational age (SGA) at discharge.

In the overall sample, GQ scores declined significantly over time by −6.34, −8.54, −12.38, −20.48, and −20.00 points at 6-, 9-, 12-, 18-, and 24-months CA, respectively. GQ declines were steeper at each time point among newborns from low HDI migrant mothers compared with those born from Italian mothers, reaching a gap of −18.54 points at 24-months CA after they started with similar values at 3-months. Still, in the low HDI migrant group, mixed milk feeding conferred a significant advantage over exclusively formula feeding at 9-, 18-, and 24-months CA and results were in the same direction for human milk feeding. The nine estimated GQ trajectories by HDI category and diet are provided in Figure 3; eight of them fell in the normal or mild delay range at 24-months, whereas the GQ trajectory of exclusively formula-fed infants born from low HDI migrant mothers declined to a score of 73.8 at 24-months, denoting moderate delay.

**Figure 4:**
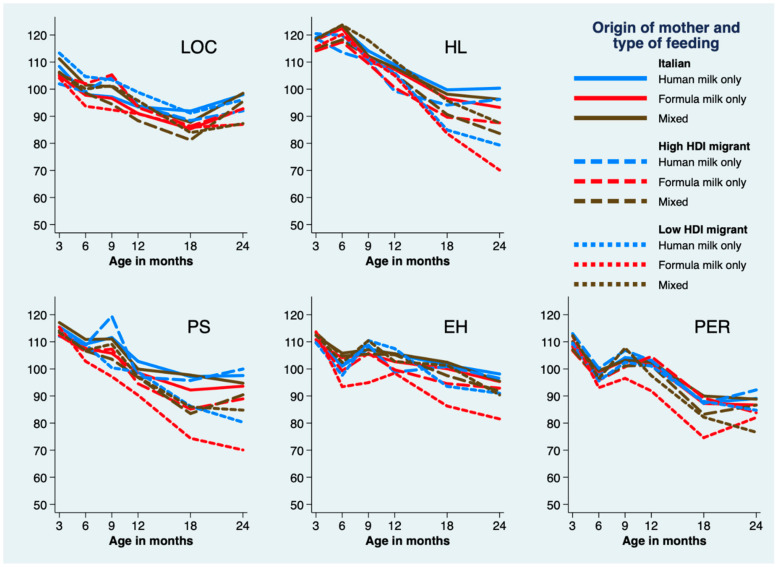
Trajectories of GMDS-R domain subscales estimated by using mixed-effects modeling, by mother’s origin and type of milk feeding and adjusted for mother’s smoking status, and infant IVH/PVL, sepsis, BPD, mechanical ventilation, and SGA at discharge. LOC, locomotor scale; HL, Hearing and Language scale; PS, Personal-social scale; EH, Eye-Hand coordination scale; PER, Performance scale.

The trajectories of the locomotor subscale were overlapping in the nine groups (Figure 4). On the contrary, the hearing–language trajectories changed according to the HDI but were unrelated with the type of feeding. In particular, the trajectories of infants from low HDI migrant mothers showed a significantly steeper decline at 18- (b = −10.33, *p* = 0.026) and 24-months (b = −20.62, *p* = 0.001) than that of infants born from Italian mothers. 

The trajectories of personal–social, eye–hand coordination, and performance subscales, varied according to mothers’ origin and diet. Low HDI exclusively formula-fed infants had lower scores on all these three subscales starting from 6-months. Eye–hand and personal–social scores progressively declined and never caught up, whereas their performance scores recovered at 24-months. See Supplementary File, Table S3 for the detailed Stata output.

## 4. Discussion

Our results indicate that early exposure to human milk and mixed milk has a beneficial effect on neurodevelopmental trajectories of preterm newborns, especially in those of migrant mothers originating from low HDI countries. These findings are consistent with Patra et al. [29] who reported better neurodevelopmental outcomes at 20-months among very low-birthweight infants fed human milk at increasing doses during NICU stay. Another study reported no evidence of an association between >50% breast milk feeding during the first 28-days of life and 2-year neurodevelopment, after adjusting for social risk factors [30]. 

The developmental disadvantage found in the present study in infants born from low HDI migrant mothers was associated with the higher maternal social risk score in this group, especially in low HDI migrant mothers, consistent with other studies showing an adverse effect of family and maternal social risk factors on preterm children’s cognitive outcomes [19,31].

Van Veen et al. [32] reported a cognitive disadvantage in multilingual compared to monolingual very and extremely preterm infants at 2-years of age, after adjusting for gestational age or parental education, supporting the hypothesis that multilingualism negatively impacts on cognitive outcomes in vulnerable preterm infants, probably because of an overload of information. However, these authors did not take migrant status into account. Our findings bring new evidence concerning preterm infants’ cognitive development highlighting delayed eye–hand coordination and performance subscale scores already from 6-months CA in preterm infants from low HDI migrant mothers and exclusively formula-fed, supporting the hypothesis that nonverbal cognitive development is affected both by low HDI maternal migrant condition and feeding. Since eye–hand coordination and performance are basic abilities, developing from the first months of life and providing infants with opportunities for acting in the world, gathering information, and learning, they may play a crucial role in the development of complex cognitive and language abilities [33,34]. Personal–social scores of this group of infants declined progressively and diverged remarkably from those of the other preterm infants in the second year of age. Since different cultural practices in the personal–social domain, i.e., reaching autonomy in feeding, dressing, and daily adaptive behaviors and socializing with caregivers and people, have been observed in Western industrialized cultures vs. African and Asian rural cultures [35], it is unclear whether the differences observed in this study reflected a delay and/or cultural differences. Migrant mothers’ cultural practices should thus be further investigated to understand infants’ personal–social achievements. Language trajectories were delayed in infants born from migrant mothers, regardless of the type of feeding, with a much steeper decline in the second year of life in infants from low HDI mothers. However, it should be noted the language scale focused on the acquisition of the receiving-country language, whereas mastery of their mother tongue could not be examined. In order to fully understand the level of language acquisition in these infants, the duration and quantity of exposure to the language of the migrant mother and that of the receiving-country should be investigated [36]. Lastly, locomotor developmental trajectories were unrelated to mother’s origin or feeding. Gross-motor functions were mainly affected by medical complications associated with preterm birth, especially by IVH/PVL. Thus, even if motor educational practices vary across cultures [35], no differences were found across trajectories, given that the incidence of complications was unrelated with mothers’ origin and feeding.

### Limitations and Future Research 

Our findings should be interpreted in light of some limitations. Information on family social background, years of stay in Italy, mastery of the Italian language, and information on newborn’s father was incomplete or unavailable, as well as mothers’ characteristics such as nutrition, body mass index, and alcohol use during gestation. Information on diet was not collected after discharge, limiting our ability to ascertain the influence of nutritional patterns on developmental outcomes. The size of the low HDI migrant group is small, therefore our findings concerning the association between diet and neurodevelopment in this group require confirmation in larger samples.

Development was assessed with reference to the Griffiths English normative values, for the absence of Italian normative values. Lastly, the evaluation of the language domain was carried out in Italian, leading to a possible bias. However, since the lower development of exclusive formula-fed infants from low HDI migrant mothers was consistently found in three other subscales, we deem that our conclusions were not affected by this bias. 

## 5. Conclusions

Our findings are relevant for clinicians working in the early childhood development area and may inform health policy decisions in several ways. They highlight the importance of implementing early follow-up programs for preterm infants, particularly for those born from low HDI migrant mothers. Integrating migrant mothers in the receiving community, monitoring and supporting their pre- and post-natal physical and psychological environment, and supporting neonatal feeding with human milk or mixed milk as soon and as far as possible should be promoted in order to prevent or reduce psychomotor impairment of their infants. Further research should be conducted to fully understand the mechanisms through which, family background, maternal gestational environment, including nutrition, and type of neonatal feeding interact in influencing infant neurodevelopment, focusing on the role of family social support and mother–infant interaction in migrant families. Factors that could have neuroprotective effects on fetal and infant neurodevelopment therefore need to be further investigated, promoted, and supported in migrant mothers starting already from gestation and continuing during the post-natal period.

## Figures and Tables

**Table 1:** Characteristics of the study population by mothers’ origin.

Variables	Italy (*n* = 384)	High HDI (*n* = 87)	Low HDI (*n* = 59)	Test (*p*-Value)	Significant Post-Hoc Comparisons at *p* = 0.017
Maternal characteristics					
Age (*n* = 529) mean ± SD	34.9 ± 5.2	31.9 ± 6.1	30.1 ± 5.8	27.3; <0.001 *	Italy > High HDI, Low HDI
Education (*n* = 527)				27.1; <0.001 ^#^	
Low *n* (%)	57 (14.9)	19 (22.1)	23 (39.0)		Low HDI > Italy, High HDI
Middle *n* (%)	174 (45.6)	45 (52.3)	26 (44.1)		
High *n* (%)	151 (39.5)	22 (25.6)	10 (16.9)		Low HDI < Italy
Working condition (*n* = 529)				<0.001 ^$^	
Employed *n* (%)	350 (91.4)	53 (60.9)	26 (44.1)		Italy > High HDI, Low HDI
Unemployed *n* (%)	8 (2.1)	6 (6.9)	3 (5.1)		Italy < High HDI, Low HDI
Student *n* (%)	8 (2.1)	5 (5.7)	2 (3.4)		Italy < High HDI
Housewife *n* (%)	17 (4.4)	23 (26.4)	28 (47.5)		Italy < High HDI < Low HDI
Smoker (*n* = 423)	35 (11.7)	13 (16.9)	6 (13.0)	1.50; 0.472 ^#^	
Maternal social risk score (*n* = 529) mean ± SD	0.37 ± 0.60	0.70 ± 0.82	1.05 ± 0.84	35.5; <0.001 ^§^	Italy < High HDI < Low HDI
Neonatal characteristics					
Sex (females) *n* (%)	191 (49.7)	44 (50.6)	35 (59.3)	1.9; 0.390 ^#^	
Weight at birth (gr.) mean ± SD	1173 ± 340	1163 ± 329	1086 ± 305	1.7; 0.178 *	
Gestational age (weeks) mean ± SD	29.2 ± 2.5	28.9 ± 2.6	28.4 ± 2.3	3.2; 0.042 *	
<28 weeks *n* (%)	92 (24.0)	22 (25.3)	21 (35.6)		
28–31 weeks *n* (%)	240 (62.5)	58 (66.7)	32 (54.2)		
32–35 weeks *n* (%)	52 (13.5)	7 (8.0)	6 (10.2)		
Born in 2010–2014 *n* (%)	194 (50.5)	56 (64.4)	34 (57.6)	5.9; 0.052 ^#^	Italy < High HDI
SGA at birth *n* (%)	76 (19.8)	12 (13.8)	11 (18.6)	1.7; 0.432 ^#^	
Firstborn *n* (%)	308 (80.2)	61 (70.1)	31 (52.5)	22.8; <0.001 ^#^	Italy > Low HDI
Twins (*n* = 529) *n* (%)	152 (39.7)	29 (33.3)	14 (23.7)	6.2; 0.046 ^#^	Italy > Low HDI
IVH or PVL (*n* = 527) *n* (%)	22 (5.8)	11 (12.6)	7 (11.9)	6.5; 0.039 ^#^	Italy < Low HDI, High HDI
Mechanical ventilation *n* (%)	107 (27.9)	23 (26.4)	24 (40.7)	4.4; 0.110 ^#^	
BPD (*n* = 527) *n* (%)	78 (20.5)	22 (25.3)	18 (30.5)	3.5; 0.177 ^#^	
Sepsis (*n* = 526) *n* (%)	53 (13.9)	12 (13.8)	11 (19.0)	1.1; 0.584 ^#^	
NEC req. surgery *n* (%)	16 (4.2)	3 (3.4)	3 (5.1)	0.2; 0.888 ^#^	
Weight at discharge (*n* = 521) mean ± SD	2094 ± 397	2085 ± 376	2206 ± 441	3.0; 0.223 ^§^	
SGA at discharge (*n* = 521) *n* (%)	274 (72.9)	65 (74.7)	37 (63.8)	2.4, 0.301 ^#^	
Length of stay (days) mean ± SD	56.3 ± 33.4	59.4 ± 40.2	64.7 ± 37.7	3.7; 0.159 ^§^	
Diet at discharge				5.2; 0.270 ^#^	
Human milk only *n* (%)	112 (29.2)	16 (18.4)	14 (23.7)		
Formula milk only *n* (%)	106 (27.6)	25 (28.7)	19 (32.2)		
Mixed *n* (%)	166 (43.2)	46 (52.9)	26 (44.1)		

Note: According to the HDI (Human Development Index) quartiles, mothers’ origin was defined as Italy, high HDI (first and second quartile) and low HDI (third and fourth quartile). Italy that ranks in the first quartile, was used as reference category. Number of cases used in the analyses is reported only for variables with missing data. * Analysis of Variance with Bonferroni corrected post-hoc comparisons; ^#^ *χ*^2^ test; ^$^ Fisher’s exact test (only *p*-value reported); ^§^ Kruskal–Wallis test; HDI, Human Development Index; SD, standard deviation; SGA, small for gestational age; IVH, intraventricular hemorrhage; PVL, periventricular leukomalacia; BPD, bronchopulmonary dysplasia; NEC, necrotizing enterocolitis.

**Table 2:** Characteristics of the study population by diet at discharge.

Variables	Human Milk (*n* = 142)	Mixed (*n* = 238)	Formula Milk (*n* = 149)	Test (*p*-Value)	Significant Post-Hoc Comparisons at *p* = 0.017
Maternal characteristics					
Age (*n* = 529) mean ± SD	33.5 ± 5.5	34.3 ± 5.1	33.5 ± 6.5	1.4; 0.256	
Education (*n* = 527)				21.5; <0.001 ^#^	
Low *n* (%)	15 (10.6)	49 (20.6)	35 (23.6)		Human < Formula, Mixed
Middle *n* (%)	66 (46.8)	98 (41.2)	81 (54.7)		
High *n* (%)	60 (42.6)	91 (38.2)	32 (21.6)		Formula< Human, Mixed
Working condition (*n* = 529)				0.197 ^$^	
Employed *n* (%)	122 (85.9)	194 (81.5)	113 (75.8)		
Unemployed *n* (%)	2 (1.4)	11 (4.6)	4 (2.7)		
Student *n* (%)	3 (2.1)	5 (2.1)	7 (4.7)		
Housewife *n* (%)	15 (10.6)	28 (11.8)	25 (16.8)		
Smoker (*n* = 423)	5 (5.3)	26 (12.4)	23 (19.3)	0.42; 0.009 ^#^	Human < Formula
Maternal social risk score (*n* = 529) mean ± SD	0.35 ± 0.58	0.50 ± 0.69	0.64 ± 0.81	8.2; 0.016 ^§^	Human < Formula
Neonatal characteristics					
Sex (females) *n* (%)	71 (50.0)	125 (52.5)	74 (49.3)	0.4; 0.801 ^#^	
Birthweight (g) mean ± SD	1173 ± 339	1234 ± 308	1035 ± 340	17.3; <0.001 *	Formula < Human, Mixed
Gestational age mean ± SD	29.1 ± 2.3	29.7 ± 2.3	28.1 ± 2.6	19.7; <0.001*	Formula < Human, Mixed
<28 weeks *n* (%)	33 (23.2)	43 (18.1)	59 (39.3)		
28–31 weeks *n* (%)	95 (66.9)	153 (64.3)	82 (54.7)		
32–35 weeks *n* (%)	14 (9.9)	42 (17.6)	9 (6.0)		
Born in 2010–2014 *n* (%)	36 (25.3)	176 (73.9)	72 (48.0)	87.1; <0.001 ^#^	Human < Formula < Mixed
SGA at birth *n* (%)	24 (16.9)	43 (18.1)	32 (21.3)	1.0; 0.592 ^#^	
Firstborn *n* (%)	115 (81.0)	177 (74.4)	108 (72.0)	3.5; 0.177 ^#^	
Twins (*n* = 529) *n* (%)	57 (40.1)	98 (41.2)	40 (26.8)	9.0; 0.011 ^#^	Formula < Human, Mixed
IVH or PVL (*n* = 527) *n* (%)	7 (4.9)	15 (6.4)	18 (12.0)	6.1; 0.048 ^#^	
Mechanical ventilation *n* (%)	39 (27.5)	49 (20.6)	66 (44.0)	24.7; <0.001 ^#^	Formula > Human > Mixed
BPD (*n* = 527) *n* (%)	78 (20.5)	18 (30.5)	22 (25.3)	3.5; 0.177 ^#^	
Sepsis (*n* = 526) *n* (%)	53 (13.9)	11 (19.0)	12 (13.8)	1.1; 0.584 ^#^	
NEC requiring surgery *n* (%)	16 (4.2)	3 (5.1)	3 (3.4)	0.2; 0.888 ^#^	
Weight at discharge (*n* = 521) mean ± SD	2094 ± 397	2206 ± 441	2085 ± 376	3.0; 0.223 ^§^	
SGA at discharge (*n* = 521) *n* (%)	274 (72.9)	37 (63.8)	65 (74.7)	2.4, 0.301 ^#^	
Length of stay (days) mean ± SD	56.3 ± 33.4	64.7 ± 37.7	59.4 ± 40.2	3.7; 0.159 ^§^	

Note: Number of cases used in the analyses is reported only for variables with missing data; * Analysis of Variance with Bonferroni corrected post-hoc comparisons; ^#^ *χ*^2^ test; ^$^ Fisher’s exact test (only *p*-value reported); ^§^ Kruskal–Wallis test; SD, standard deviation; SGA, small for gestational age; IVH, intraventricular hemorrhage; PVL, periventricular leukomalacia; BPD, bronchopulmonary dysplasia; NEC, necrotizing enterocolitis.

**Table 3:** Mean scores of the revised Griffiths Mental Development Scales (GMDS-R) General Quotient at follow-up and prevalence of delayed impairment at 24-months by mother’s origin and type of feeding.

Variables	Mothers’ Origin					Type of Feeding				
	Italy (*n* = 384)	High HDI (*n* = 87)	Low HDI (*n* = 59)	ANOVA Test (F; *p*)	Significant Post-Hoc Comparisons at *p* = 0.017	Human M. (*n* = 142)	Mixed (*n* = 238)	Formula (*n* = 150)	ANOVA Test (F; *p*-value)	Significant Post-Hoc Comparisons at *p* = 0.017
3-months	114.3 ± 9.4	111.1 ± 9.6	112.6 ± 9.6	3.7, 0.024	Italy > High HDI	114.6 ± 8.0	115.3 ± 8.0	110.0 ± 11.9	13.8, <0.001	Formula < Human, Mixed
6-months	107.3 ± 12.1	103.1 ± 14.1	101.5 ± 13.8	7.3, 0.001	Italy > High HDI, Low HDI	106.0 ± 13.0	107.9 ± 11.4	102.9 ± 14.0	6.6, 0.002	Formula < Mixed
9-months	106.8 ± 12.8	105.1 ± 13.9	105.2 ± 14.8	0.7, 0.514		107.9 ± 11.1	108.0 ± 12.5	102.4 ± 15.4	7.6, 0.001	Formula < Human, Mixed
12-months	101.7 ± 13.1	98.2 ± 14.1	98.5 ± 11.5	3.1, 0.045		102.3 ± 12.8	101.9 ± 12.4	97.7 ± 14.1	5.6, 0.004	Formula < Human, Mixed
18-months	94.7 ± 13.7	87.6 ± 13.7	83.5 ± 16.6	18.4, <0.001	Italy > High HDI, Low HDI	94.8 ± 13.0	94.1 ± 13.6	87.5 ± 16.2	11.0, <0.001	Formula < Human, Mixed
24-months	94.5 ± 14.4	90.9 ± 14.4	79.8 ± 19.9	19.4, <0.001	Low HDI < Italy, High HDI	95.9 ± 13.6	93.2 ± 15.3	87.8 ± 17.1	9.0, <0.001	Formula < Human, Mixed
Delayed impairment at 24-months, *n* (%)				56.6, <0.001 *					18.9, 0.004 *	
Severe	18 (5.3)	5 (6.9)	12 (27.3)		Low HDI > High HDI, Italy	4 (3.1)	15 (7.2)	16 (13.2)		Formula > Human, Mixed
Moderate	11 (3.2)	3 (4.2)	9 (20.4)		Low HDI > High HDI, Italy	8 (6.3)	9 (4.4)	6 (5.0)		
Mild	65 (19.1)	18 (25.0)	7 (15.9)			17 (13.3)	41 (19.8)	32 (26.4)		
Normal development	246 (72.4)	46 (63.9)	16 (36.4)		Low HDI < High HDI, Italy	99 (77.3)	142 (68.6)	67 (55.4)		

* *χ*^2^: test; HDI, Human Development Index.

## Supplementary Materials

The following are available online at https://www.mdpi.com/1660-4601/17/12/4588/s1, Table S1. Classification of mothers’ country of birth by United Nations Human Development Index (HDI) quartiles and in the HDI variable used in the study. Table S2. Stata output of the mixed effects model on the revised Griffiths Mental Development Scales GMDS-R general quotient. Table S3A. Stata output of the mixed effects model on GMDS-R subscales. Locomotor Scale. Table S3B. Stata output of the mixed effects model on GMDS-R subscales. Personal–Social Scale. Table S3C. Stata output of the mixed effects model on GMDS-R subscales. Eye–Hand Coordination Scale. Table S3D. Stata output of the mixed effects model on GMDS-R subscales. Hearing–Language Scale. Table S3E. Stata output of the mixed effects model on GMDS-R subscales. Performance Scale. Figure S1. Observed neurodevelopmental trajectories of children with different ascertained weight restrictions during follow-up.

## Abbreviations

BPD, bronchopulmonary dysplasia; CA, corrected age; EH, Eye–Hand coordination; GA, gestational age; GMDS-R, revised Griffiths Mental Development Scales; G,: General developmental quotient; HDI, Human Development Index; HL, Hearing–Language; HM, human milk; IVH, intraventricular hemorrhage; LOC, Locomotor; MV, mechanical ventilation; NEC, necrotizing enterocolitis; NICU, Neonatal Intensive Care Unit; PERF, Performance; PS, Personal–Social; PVL, periventricular leukomalacia; SGA, small for gestational age.
