# Supplementary material for: Neurodevelopmental Trajectories of Preterm Infants of Italian Native-Born and Migrant Mothers and Role of Neonatal Feeding
Source: Int J Environ Res Public Health. 2020 Jun 25;17(12):4588. doi: 10.3390/ijerph17124588 (PMC7344423; doi:10.3390/ijerph17124588)
Supplement: Supplementary file 1 [file ijerph-17-04588-s001.pdf]

Table S1. Classification of mothers' country of birth by United Nation Human Development Index (HDI) quartiles and in the HDI variable used in the study.

Mothers' countries of birth	HDI quartiles	HDI variable used in the study
Italy (384)		Italy (384)
Belgium(1), France(1), Poland(3), San Marino(2), Spain(1), Hungary(1), Slovenia(2), Japan(1), Cuba(1), USA(4), Argentina(1), Chile(1)	Very High HDI (19)	High HDI (87)
Albania(3), Bulgaria(1), Romania(31), Ukraine(2), Macedonia(1), Montenegro(1), Serbia(1), Kosovo(3), Sri Lanka(1), China(3), Iran(1), Thailand(1), Algeria(1), Tunisia(4), Costa Rica(1), Dominican Republic(1), Brazil(5), Colombia(2), Ecuador(4), Peru(1)	High HDI (68)	
Moldova(7), Bangladesh(9), Philippines(5), Egypt(1), Morocco(10), Paraguay(2)	Medium HDI (34)	Low HDI (59)
Pakistan(10), Côte d'Ivoire(1), Cameroon(1), Ethiopia(4), Nigeria(6), Senegal(2), Eritrea(1)	Low HDI (25)	

Number of preterm infants is reported in brackets.

Table S2. Stata output of the mixed effects model on the GMDS-R general quotient.

```
Multiple-imputation estimates                  Imputations       =         10
Mixed-effects REML regression                  Number of obs     =      2,728

Group variable: ID                             Number of groups  =        525
                                               Obs per group:
                                                             min =          1
                                                             avg =        5.2
                                                             max =          6
                                               Average RVI       =     0.0031
                                               Largest FMI       =     0.1155
DF adjustment:   Large sample                  DF:     min       =     704.37
                                                       avg       =   5.11e+11
                                                       max       =   1.15e+13
Model F test:       Equal FMI                  F(  59, 5.3e+07)  =      27.03
                                               Prob > F          =     0.0000

------------------------------------------------------------------------------
-
              GQ |      Coef.   Std. Err.      t    P>|t|     [95% Conf.
Interval]
-----------------+------------------------------------------------------------
-
            time |
              6  |  -6.344738   1.071653    -5.92   0.000    -8.445139   -
4.244337
              9  |  -8.541277   1.156979    -7.38   0.000    -10.80891   -
6.273639
             12  |  -12.38089   1.167665   -10.60   0.000    -14.66947   -
10.09231
             18  |  -20.48051   1.373612   -14.91   0.000    -23.17274   -
17.78828
             24  |  -20.00076   1.632237   -12.25   0.000    -23.19988   -
16.80163
                 |
             HDI |
              1  |  -.7793688   2.505959    -0.31   0.756    -5.690963
4.132225
              2  |  -.0028258   2.833575    -0.00   0.999    -5.556562
5.550911
                 |
        time#HDI |
            6 1  |  -.4655794   2.440577    -0.19   0.849    -5.249022
4.317863
            6 2  |  -5.276194   2.773062    -1.90   0.057     -
10.7113     .158908
            9 1  |   2.912412   2.662663     1.09   0.274    -2.306311
8.131135
            9 2  |  -4.060886   2.965167    -1.37   0.171    -9.872507
1.750734
```

12 1	1.995152	2.746442	0.73	0.468	-3.387775	7.378079
12 2	-4.927323	3.023782	-1.63	0.103	-10.85383	.9991802
18 1	-1.908322	3.067761	-0.62	0.534	-7.921023	4.104379
18 2	-11.36322	3.62037	-3.14	0.002	-18.45901	-4.267423
24 1	-2.146221	3.758357	-0.57	0.568	-9.512465	5.220022
24 2	-18.53784	4.630796	-4.00	0.000	-27.61404	-9.461652
diet1						
human only	1.055811	1.518102	0.70	0.487	-1.919623	4.031245
mixed	2.221134	1.427456	1.56	0.120	-.5766479	5.018917
time#diet1						
6#human only	-1.244334	1.469304	-0.85	0.397	-4.124117	1.635448
6#mixed	-.0647102	1.372535	-0.05	0.962	-2.75483	2.62541
9#human only	1.782018	1.566365	1.14	0.255	-1.288001	4.852037
9#mixed	.6302739	1.484913	0.42	0.671	-2.280101	3.540649
12#human only	1.616673	1.601212	1.01	0.313	-1.521646	4.754992
12#mixed	-.5060022	1.490278	-0.34	0.734	-3.426893	2.414889
18#human only	2.130728	1.897643	1.12	0.262	-1.588584	5.85004
18#mixed	.86629	1.755198	0.49	0.622	-2.573834	4.306414
24#human only	3.17308	2.236564	1.42	0.156	-1.210504	7.556664
24#mixed	-.5942361	2.075621	-0.29	0.775	-4.662379	3.473907
HDI#diet1						
1#human only	-2.887416	3.931479	-0.73	0.463	-10.59298	4.818143
1#mixed	-2.636768	3.135723	-0.84	0.400	-8.782685	3.509149
2#human only	.4494644	4.214942	0.11	0.915	-7.811676	8.710604
2#mixed	-.8150445	3.700253	-0.22	0.826	-8.067452	6.437363
time#HDI#diet1						
6#1#human only	2.419042	3.813126	0.63	0.526	-5.054548	9.892632
6#1#mixed	-.4846477	3.071944	-0.16	0.875	-6.505547	5.536251
6#2#human only	3.805156	4.210278	0.90	0.366	-4.446837	12.05715
6#2#mixed	2.418798	3.626403	0.67	0.505	-4.68882	9.526417
9#1#human only	2.27323	4.154013	0.55	0.584	-5.868486	10.41495
9#1#mixed	-3.777473	3.327209	-1.14	0.256	-10.29868	2.743737
9#2#human only	3.599026	4.446451	0.81	0.418	-5.115859	12.31391
9#2#mixed	7.222481	3.926727	1.84	0.066	-.4737622	14.91872

```
12#1#human only  |  -2.799876   4.230407    -0.66   0.508    -11.09132
5.49157
     12#1#mixed  |   -2.05318   3.428438    -0.60   0.549    -8.772795
4.666435
12#2#human only  |   3.702878    4.58873     0.81   0.420    -5.290867
12.69662
     12#2#mixed  |   4.526052   3.990144     1.13   0.257    -3.294486
12.34659
18#1#human only  |   2.753109   4.873975     0.56   0.572    -6.799706
12.30592
     18#1#mixed  |  -2.989632   3.893677    -0.77   0.443     -10.6211
4.641835
18#2#human only  |   3.822159   5.456894     0.70   0.484    -6.873157
14.51747
     18#2#mixed  |   7.126522   4.710379     1.51   0.130    -2.105652
16.3587
24#1#human only  |   4.087615   5.880167     0.70   0.487    -7.437301
15.61253
     24#1#mixed  |   .2018803   4.690501     0.04   0.966    -8.991333
9.395093
24#2#human only  |   7.676563   6.673235     1.15   0.250    -5.402737
20.75586
     24#2#mixed  |   8.661622   5.820847     1.49   0.137    -2.747028
20.07027
                 |
          smoker |  -3.987292   1.268059    -3.14   0.002    -6.476921   -
1.497664
         IVH_PVL |  -12.48822   1.597322    -7.82   0.000    -15.61892   -
9.357518
              MV |  -2.626645   1.020449    -2.57   0.010    -4.626693
-.626597
             BPD |  -3.891083   1.093606    -3.56   0.000    -6.034512   -
1.747654
          sepsis |  -4.090452   1.201381    -3.40   0.001    -6.445129   -
1.735774
           SGA_d |  -2.104306    .878012    -2.40   0.017    -3.825264
-.3833478
           _cons |   117.2759   1.358554    86.32   0.000     114.6131
119.9387
---------------------------------------------------------------------------------
-

------------------------------------------------------------------------------
  Random-effects Parameters  |   Estimate   Std. Err.     [95% Conf. Interval]
-----------------------------+------------------------------------------------
ID: Unstructured             |
                  sd(time) |    .573622   .0304697       .516906    .6365611
                 sd(_cons) |   8.316654   .4381604      7.500726    9.221339
           corr(time,_cons) |  -.3917204   .0590842     -.5009875   -.2701611
-----------------------------+------------------------------------------------
               sd(Residual) |   7.056268   .1238155       6.81772    7.303163
------------------------------------------------------------------------------
```

Table S3A. Stata output of the mixed effects model on GMDS-R subscales. Locomotor Scale.

```
Multiple-imputation estimates                   Imputations      =         10
Mixed-effects REML regression                   Number of obs    =      2,744

Group variable: ID                              Number of groups =        529
                                                Obs per group:
                                                              min =          1
                                                              avg =        5.2
                                                              max =          6
                                                Average RVI      =     0.0001
                                                Largest FMI      =     0.0027
DF adjustment:   Large sample                   DF:     min      = 1263375.43
                                                        avg      =   3.43e+16
                                                        max      =   4.18e+17
Model F test:       Equal FMI                   F(  57, 1.3e+11) =      13.99
                                                Prob > F         =     0.0000

--------------------------------------------------------------------------------
-
            LOC |      Coef.   Std. Err.      t    P>|t|     [95% Conf.
Interval]
----------------+---------------------------------------------------------------
-
           time |
             6  |  -7.139282   1.774355    -4.02   0.000    -10.61695    -
3.66161
             9  |  -8.290264   1.905872    -4.35   0.000    -12.02571   -
4.554823
            12  |  -13.86294   1.909692    -7.26   0.000    -17.60587   -
10.12001
            18  |  -19.57883   2.211935    -8.85   0.000    -23.91414   -
15.24352
            24  |  -12.14775   2.593789    -4.68   0.000    -17.23148   -
7.064019
                |
            HDI |
             1  |   .3462021   3.825609     0.09   0.928    -7.151853
7.844257
             2  |   -.858729   4.206073    -0.20   0.838    -9.102481
7.385023
                |
        time#HDI |
           6 1  |   3.911392   4.049143     0.97   0.334    -4.024782
11.84757
           6 2  |  -3.207714   4.490205    -0.71   0.475    -12.00835
5.592927
           9 1  |    8.32548   4.406245     1.89   0.059    -.3106022
16.96156
           9 2  |  -3.379668   4.774353    -0.71   0.479    -12.73723
5.977893
          12 1  |   1.851501   4.510261     0.41   0.681    -6.988447
10.69145
          12 2  |   .7158968    4.89376     0.15   0.884    -8.875696
10.30749
          18 1  |   .6857454   4.956212     0.14   0.890    -9.028253
10.39974
          18 2  |   1.457727   5.694912     0.26   0.798    -9.704095
12.61955
          24 1  |  -.3551598   6.006054    -0.06   0.953    -12.12681
11.41649
          24 2  |   -4.91378   7.171848    -0.69   0.493    -18.97034
9.142783
                |
          diet1 |
     human only  |    3.47794   2.281353     1.52   0.127    -.9934303
7.949309
         mixed  |   6.298741   2.149196     2.93   0.003     2.086394
10.51109
```

```
                 |
      time#diet1 |
   6#human only  |  -3.243259   2.436406    -1.33   0.183    -8.018527
1.532008
        6#mixed  |  -2.584794    2.27375    -1.14   0.256    -7.041262
1.871674
   9#human only  |  -2.927263   2.579997    -1.13   0.257    -7.983965
2.129439
        9#mixed  |  -1.935489   2.449283    -0.79   0.429    -6.735995
2.865016
  12#human only  |  -1.034866   2.621722    -0.39   0.693    -6.173348
4.103615
       12#mixed  |  -3.487032   2.439121    -1.43   0.153    -8.267621
1.293557
  18#human only  |   3.106451   3.059653     1.02   0.310    -2.890358
9.10326
       18#mixed  |   -3.84435   2.829924    -1.36   0.174    -9.390899
1.702199
  24#human only  |   1.708397   3.558246     0.48   0.631    -5.265637
8.682432
       24#mixed  |  -.5692853   3.303158    -0.17   0.863    -7.043357
5.904786
                 |
       HDI#diet1 |
   1#human only  |  -6.777543   5.898324    -1.15   0.251    -18.33805
4.78296
        1#mixed  |   -5.25821    4.77708    -1.10   0.271    -14.62112
4.104695
   2#human only  |   5.793859   6.368265     0.91   0.363     -6.68771
18.27543
        2#mixed  |  -4.386158   5.548537    -0.79   0.429    -15.26109
6.488775
                 |
   time#HDI#diet1 |
 6#1#human only  |   3.553614   6.284598     0.57   0.572    -8.763972
15.8712
      6#1#mixed  |  -1.870887    5.09694    -0.37   0.714    -11.86071
8.118932
 6#2#human only  |   4.879258   6.916061     0.71   0.481    -8.675973
18.43449
      6#2#mixed  |   7.066862   5.936764     1.19   0.234    -4.568982
18.70271
 9#1#human only  |   5.827908   6.818164     0.85   0.393    -7.535447
19.19126
      9#1#mixed  |  -9.900993   5.506724    -1.80   0.072    -
20.69397    .8919889
 9#2#human only  |   4.769839   7.269244     0.66   0.512    -9.477618
19.0173
      9#2#mixed  |   8.919631   6.399384     1.39   0.163    -3.622931
21.46219
12#1#human only  |   5.107753   6.824999     0.75   0.454    -8.268999
18.48451
     12#1#mixed  |  -2.426686   5.628144    -0.43   0.666    -13.45765
8.604273
12#2#human only  |  -.2769439   7.486873    -0.04   0.970    -14.95095
14.39706
     12#2#mixed  |   6.442035   6.503285     0.99   0.322    -6.304169
19.18824
18#1#human only  |    2.26292   7.795521     0.29   0.772    -13.01602
17.54186
     18#1#mixed  |  -2.316496   6.293707    -0.37   0.713    -14.65194
10.01894
18#2#human only  |  -7.141149   8.720925    -0.82   0.413    -24.23385
9.951551
     18#2#mixed  |   -.036194   7.508445    -0.00   0.996    -14.75247
14.68009
24#1#human only  |   .8305805   9.147917     0.09   0.928    -17.09901
18.76017
```

```
     24#1#mixed  |   2.249879   7.507141     0.30   0.764    -12.46385
16.9636
24#2#human only  |  -1.852988   10.50519    -0.18   0.860    -22.44278
18.7368
     24#2#mixed  |  -1.024116    9.15989    -0.11   0.911    -18.97717
16.92894
                 |
         IVH_PVL |  -15.96289   2.168648    -7.36   0.000    -20.21336   -
11.71242
              MV |  -4.172142   1.408028    -2.96   0.003    -6.931825   -
1.412458
             BPD |  -6.293048   1.502455    -4.19   0.000    -9.237805   -
3.348291
           SGA_d |  -3.057648   1.209028    -2.53   0.011    -5.427302
-.6879934
           _cons |   110.8231   1.971368    56.22   0.000     106.9593
114.6869
------------------------------------------------------------------------------------
-

------------------------------------------------------------------------------
  Random-effects Parameters  |   Estimate   Std. Err.     [95% Conf. Interval]
-----------------------------+------------------------------------------------
ID: Unstructured             |
                   sd(time) |   .8846789   .0487554      .7941001    .9855895
                  sd(_cons) |   11.89451   .6725826      10.64669    13.28857
           corr(time,_cons) |  -.4923195   .0549062     -.5922726   -.3774435
-----------------------------+------------------------------------------------
               sd(Residual) |   11.77377   .2033695      11.38184    12.17919
------------------------------------------------------------------------------
```

Table S3B. Stata output of the mixed effects model on GMDS-R subscales. Personal-Social Scale.

```
Multiple-imputation estimates                  Imputations       =         10
Mixed-effects REML regression                  Number of obs     =      2,728

Group variable: ID                             Number of groups  =        524
                                               Obs per group:
                                                             min =          1
                                                             avg =        5.2
                                                             max =          6
                                               Average RVI       =     0.0044
                                               Largest FMI       =     0.1909
DF adjustment:   Large sample                  DF:     min       =     263.69
                                                       avg       =   2.20e+11
                                                       max       =   2.72e+12
Model F test:       Equal FMI                  F(  59, 2.6e+07)  =      20.39
                                               Prob > F          =     0.0000

------------------------------------------------------------------------------------
-
              PS |      Coef.   Std. Err.      t    P>|t|     [95% Conf.
Interval]
-----------------+------------------------------------------------------------------
-
            time |
              6  |  -6.523389   1.591315    -4.10   0.000     -9.64231   -
3.404468
              9  |  -8.314557   1.699285    -4.89   0.000    -11.64509   -
4.984021
             12  |  -15.51657   1.676277    -9.26   0.000    -18.80201   -
12.23113
             18  |  -21.87697   1.884812   -11.61   0.000    -25.57113   -
18.18281
             24  |  -20.45363   2.149559    -9.52   0.000    -24.66668   -
16.24057
                 |
             HDI |
```

```
              1  |  -1.686026   3.110943    -0.54   0.588    -7.783365
4.411313
              2  |   1.333447   3.494656     0.38   0.703    -5.515964
8.182858
                 |
       time#HDI  |
            6 1  |   .5210669    3.61214     0.14   0.885    -6.558598
7.600732
            6 2  |  -6.064314    4.12656    -1.47   0.142    -14.15222
2.023594
            9 1  |   3.245358   3.918872     0.83   0.408    -4.435489
10.92621
            9 2  |   -9.92819   4.364094    -2.27   0.023    -18.48166    -
1.374723
           12 1  |  -2.305711   3.949371    -0.58   0.559    -10.04634
5.434915
           12 2  |  -9.590649   4.332179    -2.21   0.027    -18.08156    -
1.099734
           18 1  |  -5.299456   4.196339    -1.26   0.207    -13.52413
2.925217
           18 2  |  -19.11891   4.961597    -3.85   0.000    -28.84346    -
9.394359
           24 1  |  -2.969285   4.954364    -0.60   0.549    -12.67966
6.741089
           24 2  |  -24.86857   6.178939    -4.02   0.000    -36.97907    -
12.75808
                 |
          diet1  |
     human only  |   1.425511   1.883321     0.76   0.449    -2.265732
5.116755
          mixed  |   3.026296   1.771278     1.71   0.088    -.4453592
6.497951
                 |
     time#diet1  |
   6#human only  |   .2077452   2.185614     0.10   0.924    -4.075979
4.491469
        6#mixed  |    .287854   2.036541     0.14   0.888    -3.703692
4.2794
   9#human only  |   4.383349   2.300496     1.91   0.057    -.1255409
8.89224
        9#mixed  |   2.289723   2.179452     1.05   0.293    -1.981924
6.561371
  12#human only  |   2.809775   2.298986     1.22   0.222    -1.696154
7.315705
       12#mixed  |  -1.650678   2.136938    -0.77   0.440       -5.839
2.537644
  18#human only  |   3.530278   2.602362     1.36   0.175    -1.570258
8.630814
       18#mixed  |   2.515355    2.40664     1.05   0.296    -2.201572
7.232283
  24#human only  |   2.519921   2.938912     0.86   0.391     -3.24024
8.280082
       24#mixed  |  -1.895397   2.730412    -0.69   0.488    -7.246906
3.456111
                 |
      HDI#diet1  |
   1#human only  |  -1.747983   4.872609    -0.36   0.720    -11.29812
7.802156
        1#mixed  |  -1.307176   3.891714    -0.34   0.737    -8.934806
6.320454
   2#human only  |  -3.355127   5.233264    -0.64   0.521    -13.61214
6.901883
        2#mixed  |  -5.010261   4.574383    -1.10   0.273     -13.9759
3.955383
                 |
  time#HDI#diet1 |
 6#1#human only  |   2.617497      5.658     0.46   0.644    -8.471979
13.70697
```

```
      6#1#mixed  |  -1.750733   4.545634    -0.39   0.700    -10.66001
7.158546
 6#2#human only  |   7.898503   6.247673     1.26   0.206    -4.346712
20.14372
      6#2#mixed  |   5.751487   5.391027     1.07   0.286    -4.814731
16.31771
 9#1#human only  |   8.066167     6.1045     1.32   0.186    -3.898433
20.03077
      9#1#mixed  |  -7.738351   4.894882    -1.58   0.114    -17.33214
1.855441
 9#2#human only  |   .8017342   6.535125     0.12   0.902    -12.00688
13.61034
      9#2#mixed  |   11.66202   5.772965     2.02   0.043     .3472124
22.97682
12#1#human only  |  -.0979095   6.074539    -0.02   0.987    -12.00379
11.80797
     12#1#mixed  |   2.244936   4.923718     0.46   0.648    -7.405373
11.89525
12#2#human only  |   7.716577   6.581265     1.17   0.241    -5.182465
20.61562
     12#2#mixed  |   10.33765   5.727759     1.80   0.071    -.8885497
21.56385
18#1#human only  |   7.326048   6.676339     1.10   0.273    -5.759336
20.41143
     18#1#mixed  |  -5.924937   5.334076    -1.11   0.267    -16.37953
4.52966
18#2#human only  |   10.19782   7.490839     1.36   0.173    -4.483959
24.87959
     18#2#mixed  |   11.02858   6.470472     1.70   0.088    -1.653309
23.71047
24#1#human only  |   8.795949   7.670875     1.15   0.252     -6.23869
23.83059
     24#1#mixed  |   1.662176   6.190905     0.27   0.788    -10.47177
13.79613
24#2#human only  |   9.656661   8.852423     1.09   0.275    -7.693769
27.00709
     24#2#mixed  |   18.59837    7.76276     2.40   0.017     3.383643
33.8131
                 |
          smoker |  -3.470137   1.468933    -2.36   0.019    -6.362467
-.5778071
         IVH_PVL |  -9.679991   1.752659    -5.52   0.000    -13.11514   -
6.244839
             BPD |   -3.52203   1.093494    -3.22   0.001    -5.665239    -
1.37882
          sepsis |  -4.255378   1.303534    -3.26   0.001    -6.810263   -
1.700494
           SGA_d |  -2.428062   .9678946    -2.51   0.012    -4.325121
-.5310028
           quinq |   -3.15177    .937131    -3.36   0.001    -4.988527   -
1.315013
           _cons |   119.8464   1.687755    71.01   0.000     116.5384
123.1544
----------------------------------------------------------------------------------
-

------------------------------------------------------------------------------
  Random-effects Parameters  |   Estimate   Std. Err.     [95% Conf. Interval]
-----------------------------+------------------------------------------------
ID: Unstructured             |
                   sd(time)  |   .6622323   .0433473      .5824972    .7528819
                  sd(_cons)  |   7.736549   .6399676      6.578633    9.098272
           corr(time,_cons)  |  -.3492937   .0845689      -.503082   -.1740636
-----------------------------+------------------------------------------------
               sd(Residual)  |   10.60765    .184657      10.25183    10.97581
------------------------------------------------------------------------------
```

Table S3C. Stata output of the mixed effects model on GMDS-R subscales. Eye-Hand Coordination Scale.

```
Multiple-imputation estimates                    Imputations       =         10
Mixed-effects REML regression                    Number of obs     =      2,541

Group variable: ID                               Number of groups  =        475
                                                 Obs per group:
                                                               min =          1
                                                               avg =        5.3
                                                               max =          6
                                                 Average RVI       =     0.0055
                                                 Largest FMI       =     0.2188
DF adjustment:   Large sample                    DF:     min       =     201.90
                                                         avg       =   6.33e+11
                                                         max       =   1.10e+13
Model F test:       Equal FMI                    F(  62, 1.9e+07)  =      12.41
                                                 Prob > F          =     0.0000

-------------------------------------------------------------------------------
-
              EH |      Coef.   Std. Err.      t    P>|t|     [95% Conf.
Interval]
-----------------+-------------------------------------------------------------
-
            time |
              6  |  -5.510339   1.738407    -3.17   0.002    -8.917554   -
2.103123
              9  |   -4.26504   1.786647    -2.39   0.017    -7.766803
-.7632774
             12  |  -6.972004   1.754693    -3.97   0.000    -10.41114    -
3.53287
             18  |  -9.594176   1.826629    -5.25   0.000     -13.1743   -
6.014049
             24  |  -14.33587   1.910672    -7.50   0.000    -18.08072   -
10.59102
                 |
             HDI |
              1  |   1.155547   3.492995     0.33   0.741    -5.690604
8.001697
              2  |   4.050215   3.880017     1.04   0.297    -3.554495
11.65492
                 |
        time#HDI |
            6 1  |  -5.988614   3.939305    -1.52   0.128    -13.70951
1.732283
            6 2  |  -14.76383   4.424542    -3.34   0.001    -23.43577   -
6.091889
            9 1  |  -.8021518    4.08086    -0.20   0.844     -8.80049
7.196186
            9 2  |  -14.49355    4.53906    -3.19   0.001    -23.38995    -
5.59716
           12 1  |  -4.152214   4.093963    -1.01   0.310    -12.17623
3.871806
           12 2  |  -8.298451   4.432905    -1.87   0.061    -
16.98678    .389883
           18 1  |   -6.64392   4.060082    -1.64   0.102    -14.60153
1.313694
           18 2  |  -17.80444   4.717945    -3.77   0.000    -27.05144   -
8.557437
           24 1  |  -3.565788   4.323227    -0.82   0.409    -12.03916
4.907581
           24 2  |   -17.8318    5.54517    -3.22   0.001    -28.70013   -
6.963463
                 |
           diet1 |
```

human only	1.202736	2.129214	0.56	0.572	-2.970463	5.375935
mixed	2.685745	2.03215	1.32	0.186	-1.297232	6.668723
time#diet1						
6#human only	-4.317686	2.357185	-1.83	0.067	-8.937685	.3023119
6#mixed	-1.023223	2.222901	-0.46	0.645	-5.380029	3.333584
9#human only	.8901944	2.40567	0.37	0.711	-3.824833	5.605222
9#mixed	-1.017061	2.287853	-0.44	0.657	-5.50117	3.467048
12#human only	1.535703	2.366862	0.65	0.516	-3.103261	6.174668
12#mixed	.2326007	2.232517	0.10	0.917	-4.143052	4.608254
18#human only	.2105572	2.483214	0.08	0.932	-4.656453	5.077568
18#mixed	-.2999956	2.330451	-0.13	0.898	-4.867595	4.267604
24#human only	1.609329	2.565242	0.63	0.530	-3.418453	6.637111
24#mixed	-2.544755	2.425331	-1.05	0.294	-7.298317	2.208807
HDI#diet1						
1#human only	-1.495292	5.399983	-0.28	0.782	-12.07906	9.088481
1#mixed	-.1980436	4.391507	-0.05	0.964	-8.805248	8.409161
2#human only	-5.263405	5.942193	-0.89	0.376	-16.90989	6.383081
2#mixed	-3.915471	5.201717	-0.75	0.452	-14.11066	6.279718
time#HDI#diet1						
6#1#human only	6.557565	6.052344	1.08	0.279	-5.304811	18.41994
6#1#mixed	3.91632	4.995582	0.78	0.433	-5.874841	13.70748
6#2#human only	12.58583	6.866361	1.83	0.067	-.8719909	26.04365
6#2#mixed	11.24913	5.931187	1.90	0.058	-.3757842	22.87404
9#1#human only	2.376435	6.345778	0.37	0.708	-10.06106	14.81393
9#1#mixed	-1.704345	5.112346	-0.33	0.739	-11.72436	8.315669
9#2#human only	18.67029	6.852628	2.72	0.006	5.23939	32.1012
9#2#mixed	17.77656	6.072776	2.93	0.003	5.874139	29.67898
12#1#human only	-2.137225	6.191782	-0.35	0.730	-14.2729	9.998444
12#1#mixed	2.819651	5.164227	0.55	0.585	-7.302048	12.94135
12#2#human only	11.58123	6.810752	1.70	0.089	-1.7676	24.93006
12#2#mixed	5.296936	6.00274	0.88	0.378	-6.468217	17.06209
18#1#human only	6.401122	6.329376	1.01	0.312	-6.004227	18.80647
18#1#mixed	.7561749	5.24749	0.14	0.885	-9.528717	11.04107
18#2#human only	11.10995	7.335867	1.51	0.130	-3.268084	25.48799

```
     18#2#mixed  |   16.54904    6.28993     2.63   0.009     4.221003
28.87708
24#1#human only  |   2.286053   6.622283     0.35   0.730    -10.69338
15.26549
     24#1#mixed  |  -1.012216   5.476767    -0.18   0.853    -11.74648
9.72205
24#2#human only  |   11.92382   8.035198     1.48   0.138    -3.824878
27.67252
     24#2#mixed  |   12.63379   7.063265     1.79   0.074    -1.209951
26.47754
                 |
          smoker |  -4.328528   1.888435    -2.29   0.023    -8.052112
-.604944
                 |
           GA_cl |
          <28 w  |   .1259958   1.423254     0.09   0.929    -2.663539
2.91553
        32-35 w  |    3.34164   1.678774     1.99   0.047     .0512915
6.631988
                 |
         IVH_PVL |   -19.2095   2.090923    -9.19   0.000    -23.30769   -
15.11131
              MV |  -3.477572    1.30439    -2.67   0.008    -6.034131
-.9210126
             BPD |  -3.774626   1.539352    -2.45   0.014    -6.791703
-.757548
          sepsis |  -3.270811   1.487854    -2.20   0.028    -6.186966
-.3546568
           SGA_b |  -3.063516   1.383196    -2.21   0.027    -5.774597
-.3524355
           quinq |   2.974948   1.021697     2.91   0.004     .9724371
4.977458
           _cons |   112.1159   1.759868    63.71   0.000     108.6665
115.5653
---------------------------------------------------------------------------------
-

------------------------------------------------------------------------------
  Random-effects Parameters  |   Estimate   Std. Err.     [95% Conf. Interval]
-----------------------------+------------------------------------------------
ID: Unstructured             |
                   sd(time)  |   .3139868   .0653715      .2087818    .4722046
                  sd(_cons)  |   8.908586   .6779664      7.674114    10.34164
            corr(time,_cons) |  -.2917493   .1505491     -.5532148    .0220441
-----------------------------+------------------------------------------------
               sd(Residual)  |   11.26764    .200991      10.88051    11.66854
------------------------------------------------------------------------------
```

Table S3D. Stata output of the mixed effects model on GMDS-R subscales. Hearing-Language Scale.

```
Multiple-imputation estimates                 Imputations       =         10
Mixed-effects REML regression                 Number of obs     =      2,558

Group variable: ID                            Number of groups  =        480
                                              Obs per group:
                                                            min =          1
                                                            avg =        5.3
                                                            max =          6
                                              Average RVI       =     0.0000
                                              Largest FMI       =     0.0000
DF adjustment:   Large sample                 DF:     min       =          .
                                                      avg       =          .
                                                      max       =          .
Model F test:       Equal FMI                 F(  56,      .)   =      27.59
                                              Prob > F          =     0.0000
```

```
--------------------------------------------------------------------------------
              HL |      Coef.   Std. Err.      t    P>|t|     [95% Conf. Interval]
-----------------+--------------------------------------------------------------
            time |
              6  |   4.248814   1.420205     2.99   0.003     1.465264    7.032364
              9  |  -8.040048   1.503252    -5.35   0.000    -10.98637   -5.093729
             12  |  -10.81666   1.554688    -6.96   0.000    -13.86379   -7.769528
             18  |  -21.63306   1.826979   -11.84   0.000    -25.21387   -18.05225
             24  |  -24.85102   2.168775   -11.46   0.000    -29.10174   -20.6003
                 |
             HDI |
              1  |  -4.015628    2.78796    -1.44   0.150    -9.479929    1.448673
              2  |  -2.529788   3.028542    -0.84   0.404    -8.465621    3.406045
                 |
        time#HDI |
            6 1  |  -.9727701    3.23627    -0.30   0.764    -7.315744    5.370203
            6 2  |   .3733377   3.538693     0.11   0.916    -6.562372    7.309048
            9 1  |   3.339935   3.458124     0.97   0.334    -3.437864    10.11773
            9 2  |   3.883563    3.74079     1.04   0.299    -3.448251    11.21538
           12 1  |   -2.76902   3.647831    -0.76   0.448    -9.918637    4.380596
           12 2  |   .2570937    3.90784     0.07   0.948    -7.402132    7.91632
           18 1  |  -2.795511   4.140361    -0.68   0.500    -10.91047    5.319447
           18 2  |    -10.334   4.636604    -2.23   0.026    -19.42158   -1.246422
           24 1  |  -1.688582   4.998045    -0.34   0.735    -11.48457    8.107407
           24 2  |  -20.62066   5.960279    -3.46   0.001    -32.30259   -8.938727
                 |
           diet1 |
      human only |   .9201198   1.665827     0.55   0.581     -2.34484    4.18508
           mixed |   .3369695    1.58179     0.21   0.831    -2.763283    3.437222
                 |
      time#diet1 |
    6#human only |  -.7340741   1.930219    -0.38   0.704    -4.517234    3.049085
         6#mixed |   .8220175   1.820733     0.45   0.652    -2.746553    4.390588
    9#human only |   3.094412   2.028553     1.53   0.127    -.8814779    7.070302
         9#mixed |   1.816778   1.930479     0.94   0.347    -1.966891    5.600448
   12#human only |   .7917713   2.106322     0.38   0.707    -3.336544    4.920087
        12#mixed |   .2764081   1.985313     0.14   0.889    -3.614735    4.167551
   18#human only |   2.317272     2.4953     0.93   0.353    -2.573427    7.207971
        18#mixed |   1.313215   2.340914     0.56   0.575    -3.274893    5.901322
```

24#human only	6.186739	2.939017	2.11	0.035	.4263717	11.94711
24#mixed	2.647344	2.768938	0.96	0.339	-2.779674	8.074362
HDI#diet1						
1#human only	3.656363	4.261031	0.86	0.391	-4.695106	12.00783
1#mixed	.6471544	3.506144	0.18	0.854	-6.224762	7.519071
2#human only	3.973743	4.703432	0.84	0.398	-5.244814	13.1923
2#mixed	2.60469	4.109476	0.63	0.526	-5.449735	10.65912
time#HDI#diet1						
6#1#human only	-7.670601	4.942482	-1.55	0.121	-17.35769	2.016486
6#1#mixed	-.9969368	4.105731	-0.24	0.808	-9.044021	7.050147
6#2#human only	-4.536308	5.585462	-0.81	0.417	-15.48361	6.410997
6#2#mixed	-.2952312	4.807961	-0.06	0.951	-9.718662	9.1282
9#1#human only	-6.549845	5.332651	-1.23	0.219	-17.00165	3.90196
9#1#mixed	-.3371317	4.3365	-0.08	0.938	-8.836517	8.162253
9#2#human only	-8.068493	5.747518	-1.40	0.160	-19.33342	3.196435
9#2#mixed	1.787887	5.071442	0.35	0.724	-8.151957	11.72773
12#1#human only	-6.633601	5.436062	-1.22	0.222	-17.28809	4.020884
12#1#mixed	3.797278	4.602549	0.83	0.409	-5.223553	12.81811
12#2#human only	-4.714092	6.054608	-0.78	0.436	-16.5809	7.152721
12#2#mixed	2.084517	5.317144	0.39	0.695	-8.336894	12.50593
18#1#human only	-2.424971	6.336626	-0.38	0.702	-14.84453	9.994587
18#1#mixed	-1.352554	5.321845	-0.25	0.799	-11.78318	9.07807
18#2#human only	-5.893896	7.295854	-0.81	0.419	-20.19351	8.405716
18#2#mixed	7.891001	6.253422	1.26	0.207	-4.365481	20.14748
24#1#human only	-2.121832	7.485105	-0.28	0.777	-16.79237	12.5487
24#1#mixed	-7.545676	6.334142	-1.19	0.234	-19.96037	4.869016
24#2#human only	-1.84896	8.936965	-0.21	0.836	-19.36509	15.66717
24#2#mixed	11.78006	7.785003	1.51	0.130	-3.478265	27.03839
IVH_PVL	-5.877094	1.593762	-3.69	0.000	-9.000809	-2.753378
MV	-2.922731	1.00793	-2.90	0.004	-4.898237	-.9472246
BPD	-4.245019	1.076905	-3.94	0.000	-6.355714	-2.134324
_cons	120.2626	1.289774	93.24	0.000	117.7347	122.7905

```
  Random-effects Parameters  |   Estimate   Std. Err.     [95% Conf. Interval]
-----------------------------+------------------------------------------------
ID: Unstructured             |
                  sd(time) |   .7373241    .039512      .6638102    .8189793
                 sd(_cons) |   7.887254    .534972      6.905435    9.008669
           corr(time,_cons) |  -.5861932   .0507578     -.6769221   -.4779177
-----------------------------+------------------------------------------------
              sd(Residual) |   9.153659   .1627479      8.840173    9.478262
------------------------------------------------------------------------------
```

Table S3E. Stata output of the mixed effects model on GMDS-R subscales. Performance Scale.

```
Multiple-imputation estimates                   Imputations       =         10
Mixed-effects REML regression                   Number of obs     =      2,728

Group variable: ID                              Number of groups  =        524
                                                Obs per group:
                                                              min =          1
                                                              avg =        5.2
                                                              max =          6
                                                Average RVI       =     0.0030
                                                Largest FMI       =     0.1281
DF adjustment:   Large sample                   DF:     min       =     574.93
                                                        avg       =   1.69e+12
                                                        max       =   5.39e+13
Model F test:       Equal FMI                   F(  59, 5.7e+07)  =      17.61
                                                Prob > F          =     0.0000

-------------------------------------------------------------------------------
-
            PER |      Coef.   Std. Err.      t    P>|t|     [95% Conf.
Interval]
----------------+--------------------------------------------------------------
-
           time |
             6  |  -9.180355   1.791266    -5.13   0.000    -12.69117   -
5.669538
             9  |  -5.144685   1.907571    -2.70   0.007    -8.883456   -
1.405914
            12  |  -6.257238   1.876498    -3.33   0.001    -9.935106   -
2.579371
            18  |  -20.92748   2.092559   -10.00   0.000    -25.02882   -
16.82614
            24  |   -21.4682   2.367096    -9.07   0.000    -26.10762   -
16.82878
                |
            HDI |
             1  |   -1.45117   3.564192    -0.41   0.684     -8.43686
5.53452
             2  |   3.942099   3.995654     0.99   0.324    -3.889242
11.77344
                |
       time#HDI |
           6 1  |  -1.731948   4.068807    -0.43   0.670    -9.706664
6.242767
           6 2  |  -9.858102   4.641892    -2.12   0.034    -18.95604
-.7601611
           9 1  |  -.8217881   4.395825    -0.19   0.852    -9.437447
7.793871
           9 2  |  -10.44458   4.894877    -2.13   0.033    -20.03836
-.8508013
          12 1  |    4.11324   4.419182     0.93   0.352    -4.548197
12.77468
          12 2  |  -13.98685   4.849699    -2.88   0.004    -23.49209   -
4.481615
```

18 1	3.472083	4.661838	0.74	0.456	-5.664951	12.60912
18 2	-16.5645	5.495365	-3.01	0.003	-27.33522	-5.793783
24 1	-1.429385	5.463213	-0.26	0.794	-12.13709	9.278314
24 2	-8.578553	6.781107	-1.27	0.206	-21.86928	4.712173
diet1						
human only	.7451272	2.151005	0.35	0.729	-3.470768	4.961022
mixed	1.929213	2.02017	0.95	0.340	-2.030255	5.88868
time#diet1						
6#human only	-1.673618	2.458761	-0.68	0.496	-6.492702	3.145466
6#mixed	-1.548859	2.292024	-0.68	0.499	-6.041144	2.943426
9#human only	.3915787	2.581462	0.15	0.879	-4.667994	5.451151
9#mixed	-1.794621	2.446428	-0.73	0.463	-6.589532	3.00029
12#human only	.5348363	2.571538	0.21	0.835	-4.505285	5.574958
12#mixed	-1.233692	2.391857	-0.52	0.606	-5.921645	3.454261
18#human only	-.5675791	2.885983	-0.20	0.844	-6.224001	5.088843
18#mixed	.831351	2.670899	0.31	0.756	-4.403514	6.066216
24#human only	1.660473	3.232817	0.51	0.608	-4.675732	7.996677
24#mixed	.220383	3.005537	0.07	0.942	-5.670361	6.111127
HDI#diet1						
1#human only	5.568765	5.581041	1.00	0.318	-5.369876	16.5074
1#mixed	-1.555033	4.455832	-0.35	0.727	-10.28831	7.178241
2#human only	-3.368131	5.997141	-0.56	0.574	-15.12231	8.386049
2#mixed	-1.791203	5.228884	-0.34	0.732	-12.03964	8.457229
time#HDI#diet1						
6#1#human only	-.2651794	6.36926	-0.04	0.967	-12.7487	12.21834
6#1#mixed	1.558541	5.120152	0.30	0.761	-8.476772	11.59385
6#2#human only	6.810922	7.032405	0.97	0.333	-6.972339	20.59418
6#2#mixed	4.873782	6.065874	0.80	0.422	-7.015113	16.76268
9#1#human only	-.609376	6.850188	-0.09	0.929	-14.0355	12.81675
9#1#mixed	1.762962	5.491628	0.32	0.748	-9.000431	12.52635
9#2#human only	8.148438	7.332944	1.11	0.266	-6.223868	22.52074
9#2#mixed	12.88878	6.477998	1.99	0.047	.192134	25.58542
12#1#human only	-8.375271	6.797256	-1.23	0.218	-21.69765	4.947106
12#1#mixed	-2.349951	5.509963	-0.43	0.670	-13.14928	8.449379

```
12#2#human only  |   11.29171   7.362121     1.53   0.125    -3.137783
25.7212
     12#2#mixed  |   6.756601   6.408751     1.05   0.292     -5.80432
19.31752
18#1#human only  |  -7.748727    7.41239    -1.05   0.296    -22.27674
6.779291
     18#1#mixed  |  -7.209498   5.921395    -1.22   0.223    -18.81522
4.396223
18#2#human only  |   16.60658   8.291909     2.00   0.045     .3547324
32.85842
     18#2#mixed  |   6.606137    7.16773     0.92   0.357    -7.442355
20.65463
24#1#human only  |   .4393114   8.450953     0.05   0.959    -16.12425
17.00287
     24#1#mixed  |   2.213861   6.821804     0.32   0.746    -11.15663
15.58435
24#2#human only  |   3.629695   9.713155     0.37   0.709    -15.40774
22.66713
     24#2#mixed  |  -5.799049   8.522469    -0.68   0.496    -22.50278
10.90468
                 |
          smoker |  -3.525786    1.46704    -2.40   0.017    -6.407196
-.644375
         IVH_PVL |   -16.1696   1.801439    -8.98   0.000    -19.70037   -
12.63884
             BPD |  -4.710488   1.120371    -4.20   0.000    -6.906376     -
2.5146
          sepsis |  -5.406429   1.336369    -4.05   0.000     -8.02567   -
2.787187
           SGA_b |  -3.093037   1.110121    -2.79   0.005    -5.268853
-.9172205
           quinq |   3.387642   .9557336     3.54   0.000     1.514423
5.260861
           _cons |   110.2646   1.704801    64.68   0.000     106.9233
113.606
---------------------------------------------------------------------------------
-

------------------------------------------------------------------------------
  Random-effects Parameters  |   Estimate   Std. Err.     [95% Conf. Interval]
-----------------------------+------------------------------------------------
ID: Unstructured             |
                  sd(time) |   .7078768   .0481866       .619462    .8089109
                 sd(_cons) |   9.784651   .6985494      8.506993     11.2542
           corr(time,_cons) |  -.5980789   .0558781     -.6965981   -.4774225
-----------------------------+------------------------------------------------
               sd(Residual) |   11.92592   .2068873      11.52724    12.33838
------------------------------------------------------------------------------
```

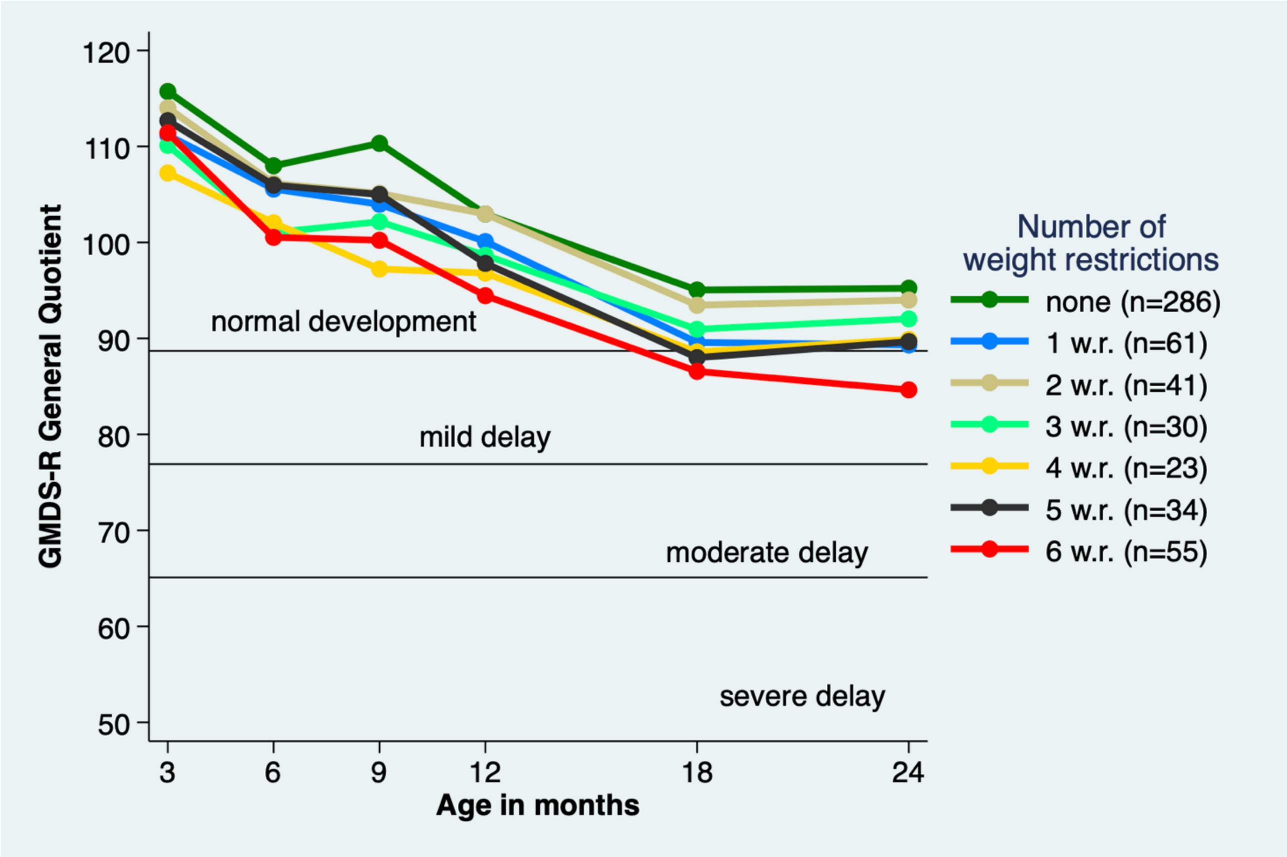

Figure S1. Observed neurodevelopmental trajectories of children with different ascertained weight restrictions during follow-up.

This figure describes the neurodevelopmental trajectories of children according to the number of times in which, in the six follow-up visits, they were found weight restricted.
